# Bioactive Phytochemical Proanthocyanidins Inhibit Growth of Head and Neck Squamous Cell Carcinoma Cells by Targeting Multiple Signaling Molecules

**DOI:** 10.1371/journal.pone.0046404

**Published:** 2012-09-26

**Authors:** Ram Prasad, Santosh K. Katiyar

**Affiliations:** 1 Birmingham Veterans Affairs Medical Center, Birmingham, Alabama, United States of America; 2 Department of Dermatology, University of Alabama at Birmingham, Birmingham, Alabama, United States of America; 3 Comprehensive Cancer Center, University of Alabama at Birmingham, Birmingham, Alabama, United States of America; 4 Nutrition Obesity Research Center, University of Alabama at Birmingham, Birmingham, Alabama, United States of America; University of Nebraska Medical Center, United States of America

## Abstract

Despite advances in surgical and medical therapies, approximate 50% survival rate of head and neck squamous cell carcinoma (HNSCC) has had marginal improvement in the last 30 years. Therefore, alternative strategies are required for the management of HNSCC. Here, we report the chemotherapeutic effect of proanthocyanidins on HNSCC cells using *in vitro* and *in vivo* models. Treatment of human HNSCC cell lines from different sub-sites, such as oral cavity (SCC1), larynx (SCC5), tongue (OSC19) and pharynx (FaDu), with grape seed proanthocyanidins (GSPs) reduced their cell viability and induced cell death in a dose- and time-dependent manner. GSPs induced inhibition of cell viability was associated with: (i) G1-phase arrest, (ii) inhibition of expressions of cyclins (cyclin D1 and Cyclin D2) and cyclin-dependent kinases (Cdk), (iii) increased expression of the Cdk inhibitory proteins (Cip1/p21, Kip1/p27), enhanced binding of Cdk inhibitors to Cdks, and downregulation of E2F transcription factor. GSPs significantly (*P*<0.05−0.001) increased apoptosis of SCC1 and OSC19 cells with induction of Bax, reduced expression of Bcl-2, and activation of caspase-3. GSPs also reduced the expression of epidermal growth factor receptor (EGFR), and treatment of SCC1 cells with erlotinib, an EGFR-targeting small molecule tyrosine kinase inhibitor, significantly (*P*<0.05−0.001) reduced cell viability and increased cell death. Dietary administration of GSPs (0.5%, w/w) in supplementation with AIN76A control diet inhibited the growth of SCC1 tumor xenografts in athymic nude mice, which was associated with: (*i*) inhibition of cell proliferation, (*ii*) induction of apoptosis of tumor xenograft cells, (iii) decreased expression of cyclins and Cdks, (*iv*) decreased expression of EGFR, and (*v*) increased expression of Cip1/p21 and Kip1/p27 proteins and their increased binding to Cdks in tumor xenograft samples. Together, these results suggest that GSPs may be a promising candidate for head and neck squamous cell carcinoma therapy.

## Introduction

Head and neck squamous cell carcinoma (HNSCC) is devastating and a challenging clinical problem due to the persisting high rate of this malignancy. In addition to high rates of occurrence, HNSCC is associated with frequent formation of second primary tumor among the highest for any malignancy [1]. HNSCC affects more than 40,000 people and responsible for approximately 20,000 deaths annually in the United States [2]. Despite advances in conventional therapies, including surgery, radiation and chemotherapy, the overall survival rate for HNSCC has had marginal improvement in the last three decades [3]–[5]. Current medical and surgical treatment options result in considerable impairment of speech and swallowing functions [6]–[9]. HNSCC, therefore, is an appropriate target for chemoprevention. If any chemopreventive agent is non-toxic and devoid of harmful side effects, it may be considered for use in primary prevention of HNSCC or may serve as appropriate therapy for patients who have had HNSCC and are at high risk for recurrence. Epidermal growth factor receptor (EGFR) is overexpressed in approximately 90% of HNSCC tumors and its overexpression is significantly associated with poor prognosis [10]–[12]. It shows that EGFR is a promising target for the treatment of HNSCC. One approved HNSCC therapy (cetuximab, an FDA approved drug for the treatment of HNSCC) and various investigational therapies (*e.g*., use of small molecule inhibitors, such as erlotinib) target EGFR. However, their poor response rates, toxicity and resistance have limited their use as clinical therapeutic agents for the majority of tumors [13], [14]. Therefore, investigations on less toxic and less resistance-associated alternative strategies are urgently needed.

Bioactive phytochemicals that are non-toxic at effective doses offer promising options for the development of effective chemotherapeutic or adjuvant therapy for conventional cytotoxic therapies. Grapes (*Vitis vinifera*) are one of the most widely consumed fruits world-wide and have enormous health benefits. The seeds of grapes are rich in proanthocyanidins with 60–70% of the proanthocyanidins being contained in the seeds. The grape seed proanthocyanidins (GSPs) are composed mainly of dimers, trimers, tetramers and oligomers of monomeric catechins [15], [16]. GSPs have been shown to have cytotoxic effects on tumor cells without having adverse effects on normal cells [17]. These bioactive phytochemicals, GSPs, have shown anti-carcinogenic effects in some animal tumor models with no apparent signs of toxicity in these animal models [18], [19]. GSPs inhibit ultraviolet radiation- and chemical carcinogen-induced skin tumor development in different mouse models [18], [20]. GSPs supplemented AIN76A control diet resulted in a dose-dependent inhibition of the growth of non-small cell lung cancer and breast cancer tumor xenografts [21], [22], and spontaneous development of prostate cancer in TRAMP mouse model [23]. However, the anti-carcinogenic potential of GSPs against HNSCC is largely unexplored.

In the current study, we assessed the chemotherapeutic effect of GSPs on HNSCC cell lines derived from different sub-sites, such as the oral cavity (UM-SCC1), larynx (UM-SCC5), pharynx (FaDu) and tongue (OSC19). To verify the observations obtained in *in vitro* model, we also assessed the effect of dietary GSPs on *in vivo* tumor xenograft growth of SCC1 cells using athymic nude mice. Erlotinib, an EGFR-targeting small molecule tyrosine kinase inhibitor is currently under clinical evaluation in HNSCC trials [24]. Therefore, we used erlotinib in our studies as a positive control. Our results show that treatment of HNSCC cell lines from different sub-sites with GSPs results in inhibition of cell proliferation/growth and induction of apoptosis and that GSPs-induced inhibition of HNSCC cell growth is mediated through a process that involves a reduction in the levels of EGFR and reactivation of cyclin-dependent kinase inhibitory (Cdki) proteins (Cip1/p21 and Kip1/p27) in HNSCC cells *in vitro* and *in vivo* models.

## Materials and Methods

### Chemicals, reagents and antibodies

The purified GSPs were received from the Kikkoman Company, Noda, Japan (no financial conflict of interest). Quality control of the GSPs is maintained by the company on lot-to-lot basis. The GSPs contain approximately 89% proanthocyanidins, with dimers (6.6%), trimers (5.0%), tetramers (2.9%) and oligomers (74.8%), as described earlier [18], [19]. MTT (3-[4, 5-dimethyl-2-yl]-2, 5-diphenyl tetrazolium bromide), erlotinib and all other chemicals were of analytical grade and purchased from Sigma Chemical Co. (St. Louis, MO). The Annexin V-conjugated AlexaFluor488 Apoptosis Detection Kit was purchased from Molecular Probes, Inc. (Eugene, OR). The protein assay kit was from Bio-Rad (Hercules, CA). The primary antibodies were obtained as follows: antibodies specific for Bax, Bcl-2, Bcl-xl, PCNA, cleaved caspase-3, EGFR, ERK1/2, p-ERK1/2, cyclin D1, cyclin D2, Cdk4, Cdk6, PARP and β-actin were purchased from Cell Signaling Technology (Beverly, MA); Rb, pRb and E2F were obtained from BD Pharmingen. Cdk2, cytochrome c, Cip1/p21, Kip1/p27, PCNA and the secondary antibodies conjugated with horseradish peroxidase were purchased from Santa Cruz Biotechnology, Inc. (Santa Cruz, CA).

### Cell lines and cell culture conditions

HNSCC cell lines generated from the oral cavity (UM-SCC1), larynx (UM-SCC5), pharynx (FaDu) and tongue (OSC19) were used in this study. FaDu and the normal (non-malignant) human bronchial epithelial cells (BEAS-2B) were procured from the American Type Culture Collection. The cell lines, UM-SCC1, UM-SCC5 and OSC19 were kindly provided by Dr. Rosenthal, University of Alabama at Birmingham, Birmingham, AL. The origin of these cell lines was University of Michigan (UM-SCC1 and UM-SCC5) and University of Texas, MD Anderson (OSC19), as detailed earlier [25]. The cells were cultured as monolayers in DMEM supplemented with 10% heat-inactivated fetal bovine serum and 100 mg/mL penicillin-streptomycin (Invitrogen), and kept in a humidified atmosphere of 5% CO_2_ at 37°C. Cells were plated in culture plates and allowed to adhere for 24 h before treatment with GSPs or erlotinib. The GSPs and erlotinib were dissolved in a small amount (50 µL) of dimethylsulfoxide (DMSO), which was added to the complete cell culture medium. The maximum concentration of DMSO in media was 0.1% (v/v). Cells treated with DMSO only served as a vehicle control.

### Cell viability assays

The effect of GSPs on the viability of HNSCC cells or normal human bronchial epithelial cells was determined using MTT assay as described previously [26]. Briefly, 1×10^4^ cells per well in a 96-well plate were treated with varying concentrations of GSPs or erlotinib for 24 and 48 h. At the end of incubation time, cells were washed with PBS buffer and further incubated with 50 µL of 5 mg/mL MTT and the resulting formazan crystals were dissolved in 150 µL of DMSO. The color absorbance was recorded at 540 nm using a Bio-Rad 3350 microplate reader. The effect of GSPs or erlotinib on cell viability was calculated in terms of percent of control, which was arbitrarily assigned a value of 100% viability.

GSPs-induced cytotoxicity also was determined using a trypan blue dye exclusion cell death assay, as described previously [26]. Briefly, 5×10^4^ cells were treated with or without GSPs (0, 10, 20, 40 and 60 µg/mL) for 24 and 48 h. Thereafter, cells were harvested, treated with 0.25% trypan blue dye and the cells that had taken up the dye were counted under a microscope using a hemocytometer. The GSPs- or erlotinib-induced cell death is expressed as the mean±SD percentage of dead cells in each treatment group from three independent experiments.

### Cell cycle phase distribution analysis

For cell cycle distribution analysis, SCC1 and OSC19 cells were treated with different concentrations of GSPs (0, 20, 40 and 60 µg/mL) in complete medium for 48 h. The cells were then harvested, and processed for cell cycle analysis, as detailed previously [26]. Briefly, the 1×10^5^ cells were re-suspended in 50 µL cold PBS to which 450 µL cold methanol was added and the cells were then incubated for 1 h at 4°C. After centrifugation, the pellet was incubated with RNase A (20 µg/mL) for 30 min. The cells were incubated with propidium iodide (50 µg/mL) on ice in the dark. The cell cycle distribution of the cells was then determined using a FACS Calibur instrument (BD Biosciences, San Jose, CA) equipped with CellQuest 3.3 software.

### Analysis of apoptotic cell death

GSPs-induced apoptotic cell death of HNSCC cells was quantitatively determined by flow cytometry using the Annexin V-conjugated Alexa fluor488 Apoptosis Detection Kit following the manufacturer's protocol, as previously described [26]. Briefly, after treatment of cells with GSPs for 48 h, cells were harvested, washed with PBS buffer and incubated with Annexin V Alexa fluor488 (Alexa488) and propidium iodide for 10 min in the dark. The stained cells were then analyzed by fluorescence activated cell sorting (FACS) using the FACS Calibur instrument (BD Biosciences) and CellQuest 3.3 software at the UAB Comprehensive Cancer Center core facility.

### Immunoblotting and Immunoprecipitation

Following treatment of HNSCC cells with or without various concentrations of GSPs or erlotinib the cells were harvested, washed with cold PBS buffer, and lysed with ice-cold lysis buffer supplemented with cocktail of protease inhibitors as detailed previously [26]. For western blot analysis, proteins were resolved on 8–10% Tris-glycine gels and transferred onto a nitrocellulose membrane. After blocking the non-specific binding sites, the membrane was incubated with the primary antibody at 4°C overnight. The membrane was then incubated with the appropriate secondary antibody and the immunoreactive protein bands were visualized using enhanced chemiluminescence reagents (Amersham Biosciences, Piscataway, NJ). The membrane was then stripped and re-probed with anti-β-actin antibody to verify equal protein loading on the gel. Blots were stripped and reprobed up to three times as needed.

For the binding assays of Cdks and Cdki (Cip1/p21, Kip1/p27), cells were treated with vehicle or GSPs (60 µg/mL) for 48 h, washed with ice-cold PBS buffer, and whole cell lysates prepared, as described previously [26], [27]. Aliquots containing 200 µg of protein were cleared with protein A/G-plus agarose beads (Santa Cruz, CA). Cip1/p21 and Kip1/p27 proteins were immunoprecipitated from whole cell lysates using specific antibodies after incubation for 8 h followed by the addition of protein A/G-plus agarose beads (50 µL, Santa Cruz, CA) and continued incubation overnight at 4°C. Immunoprecipitates were washed, and subsequently subjected to electrophoresis on 12% Tris-glycine gel followed by immunoblotting using Cdk2, Cdk4 and Cdk6 antibodies.

### Athymic nude mice and tumor xenograft study

Four-five weeks old female athymic nude mice were purchased from the National Cancer Institute (Bethesda, MD) and housed in the Animal Resource Facility at the University of Alabama at Birmingham in accordance with the Institutional Animal Care and Use Committee guidelines. All mice were maintained under standard conditions of a 12 h dark/12 h light cycle, a temperature of 24±2°C, and relative humidity of 50±10%. The mice were given control AIN76A diet with or without supplementation with GSPs (0.5%, w/w) and drinking water *ad libitum* throughout the experiment. The animal protocol used in this study was approved by the Institutional Animal Care and Use Committee of the University of Alabama at Birmingham. Approved animal protocol number is: 101109267. To determine the *in vivo* chemotherapeutic efficacy of dietary GSPs against head and neck cancer tumor xenograft growth, exponentially growing SCC1 cells (5×10^6^ in 100 µL PBS) were injected subcutaneously in the right flank of each mouse. One day after tumor cell inoculation, mice were divided randomly into two groups with eight mice per group. One group of mice received the AIN76A control diet, while the second group of mice received a 0.5% GSPs-supplemented AIN76A control diet in pellet throughout the experiment period. The experiment was terminated on 35^th^ day after tumor cell inoculation. The tumor size and body weight per mouse per week was recorded. Tumor volumes were calculated using the hemiellipsoid model formula: tumor volume = ½ (4π/3) (l/2) (w/2) h, where l = length, w = width and h = height. At the termination of the experiment, mice were sacrificed after CO_2_ gas inhalation followed by cervical dislocation, and tumor from each mouse was excised and the wet weight of each tumor in each group was recorded. A part of the tumor tissue was used to prepare tumor lysates for western blot analysis and the other part of the tissue was paraffin-embedded and used for immunohistochemical analysis.

### Immunohistochemical detection and analysis

Paraffin-embedded tumor sections (5 µm thick) were deparaffinized, rehydrated and then antigen retrieval procedure was carried out, as detailed previously [28]. The sections were then washed in PBS buffer and non-specific binding sites were blocked with 1% bovine serum albumin and 2% goat serum in PBS before incubation with either PCNA or anti-cleaved caspase-3 antibody. After washing, the sections were incubated with biotinylated secondary antibody followed by horseradish peroxidase-conjugated streptavidin. The sections were further incubated with 2, 4-diaminobenzidine substrate and counterstained with hematoxylin. The PCNA-positive or cleaved caspase 3-positive cells in a section were counted in at least 4–5 different fields and photographed using an Olympus microscope (Model BX40F4, Tokyo, Japan) fitted with a Q-color 5 Olympus camera.

### TUNEL assay for apoptotic index analysis

This assay was performed using DeadEnd™ Colorimetric TUNEL System Kit (Promega Corporation, USA) following the manufacturer's instructions. Briefly, after antigen retrieval, the tumor sections were fixed by incubation with cold 4% paraformaldehyde at 4°C. The sections were then incubated with terminal deoxynucleotidyl transferase recombinant (rTdT) enzyme-catalysed reaction and nucleotide mixture for 60 min at 37°C in the dark. After immersion in stop/wash buffer for 15 min at room temperature, the sections were washed with PBS buffer to remove unincorporated fluorescein-12-dUTP and the nuclei counterstained with hematoxylin. TUNEL-positive cells were examined and counted under microscope. The TUNEL-positive cells are expressed as a percentage of total cells in the microscopic field.

### Statistical analysis

The statistical significance of the difference between the values of control and treatment groups was determined by either Student *t* test or simple one-way ANOVA followed by Tukey's *post hoc* test for multiple comparisons using GraphPad Prism version 4.00 for Windows, GraphPad Software, San Diego, California, USA, www.graphpad.com. In each case, *P*<0.05 was considered statistically significant.

## Results

### GSPs inhibit cell viability and induce cell death of HNSCC cells, but do not affect normal human bronchial epithelial cells

**Figure 1:**
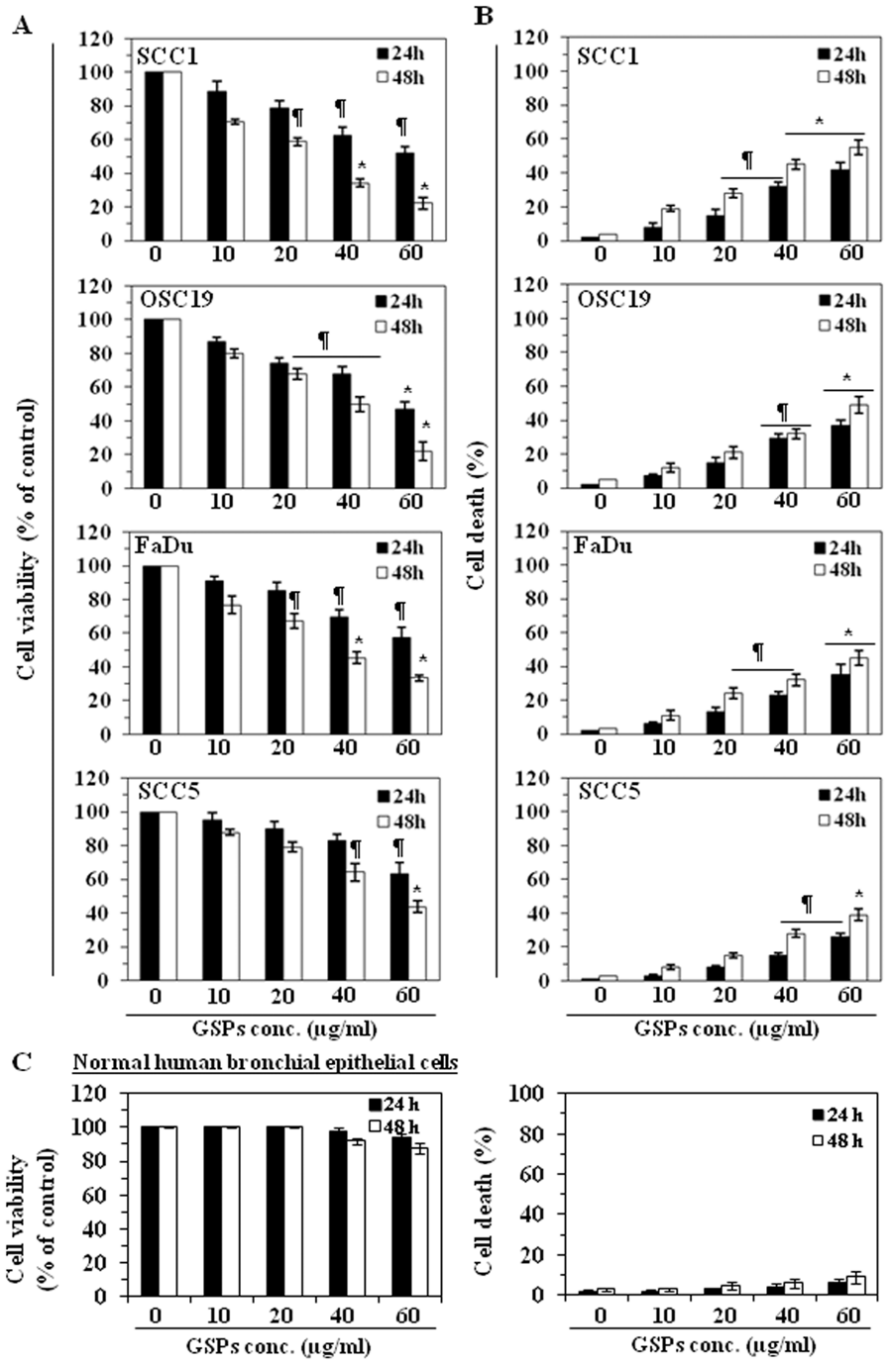
GSPs decrease cell viability and induce cell death of HNSCC cells. (**A**), Treatment of HNSCC cells (SCC1, OSC19, FaDu and SCC5) with GSPs for 24 and 48 h inhibit cell viability in a dose- and time-dependent manner. Cell viability was determined using the MTT assay as described in the Materials and Methods. The data on cell viability are expressed in terms of percent of control cells (non-GSPs treated) as the mean±SD of 8 replicates. (**B**), GSPs enhance death of HNSCC cells. Cell death was determined using the trypan blue dye exclusion assay. The cell death data are presented as the mean percent of dead cells from three independent experiments±SD *vs* control group. (**C**), Dose- and time-dependent effect of GSPs on the viability and cell death of normal human bronchial epithelial cells (BEAS-2B). Cells treated with DMSO alone as a vehicle served as a control. Significant difference *vs.* control group, ^*^ *P*<0.001; ^¶^ *P* <0.01.

The effect of GSPs on cell viability of HNSCC cell lines, SCC1, SCC5, OSC19 and FaDu, as well as normal human bronchial epithelial cells (BEAS-2B), was determined using MTT assay and cell death was assessed using trypan blue dye exclusion assay. The HNSCC cells were treated with different concentrations of GSPs (0, 10, 20, 40 and 60 µg/mL) for 24 and 48 h. A dose-dependent reduction in the viability of the SCC1 cells was observed that ranged from 8 to 50% (*P*<0.05) after 24 h, and 30 to 78% (*P*<0.01) after 48 h of treatment with GSPs, as shown in Figure 1A. Under identical conditions, similar effects of GSPs were observed on treatment of OSC19, FaDu, and SCC5 cells (Figure 1A). The effect of GSPs-induced reduction in the viability of SCC1 cells was comparatively higher than that observed for the other cell lines. We also determined the cytotoxic effect of GSPs on HNSCC cell lines in terms of cell death using the trypan blue dye exclusion assay. As shown in Figure 1B, when compared with the non-GSPs-treated control cells, treatment of SCC1 cells with GSPs resulted in a significant dose-dependent increase in cell death. Treatment of cells with GSPs for 24 h resulted in a 7–40% (*P*<0.05–*P*<0.01) increase in cell death, while treatment for 48 h resulted in 16–55% (*P*<0.05−0.001) cell death. Using identical conditions, more or less similar cytotoxic effects were observed in other HNSCC cell lines when treated with GSPs for 24 and 48 h (Figure 1B). In contrast, the sensitivity of the BEAS-2B cells to the cytotoxic effects of GSPs was much lower and could not achieve statistical significance at the highest dose of GSPs (60 µg/mL) and longer treatment times (48 h). Thus, these data suggest that GSPs do have a cytotoxic effect on HNSCC cells, but are not cytotoxic to normal human bronchial epithelial cells.

### GSPs induce G1-phase cell cycle arrest in HNSCC cells

**Figure 2:**
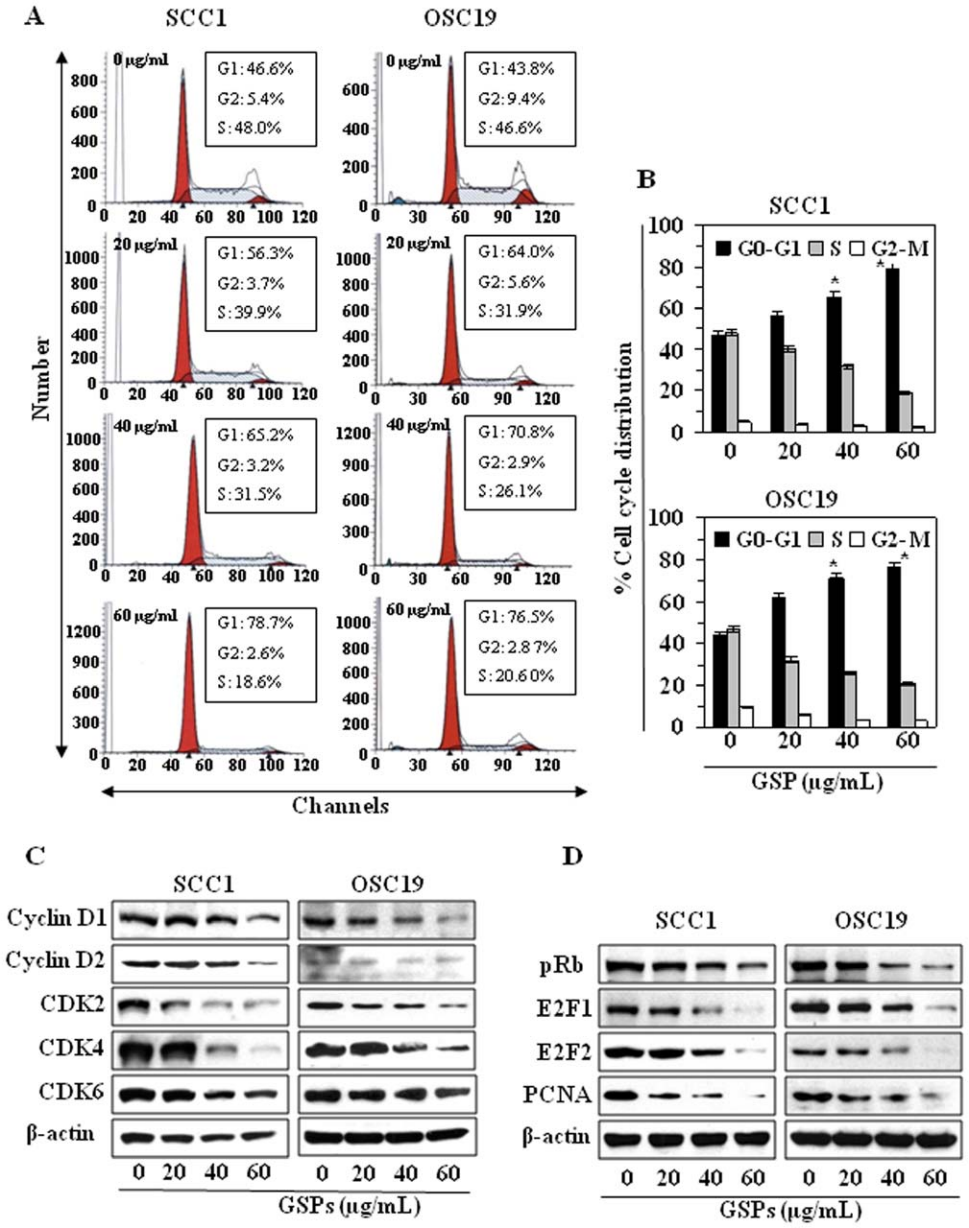
Treatment of HNSCC cells with GSPs blocks G0/G1 phase of cell cycle progression. (**A**), SCC1 and OSC19 cells were treated with either vehicle (0.1% DMSO) or GSPs (20, 40 and 60 µg/mL) in complete medium. After 48 h of treatment, cells were harvested and processed for cell cycle distribution analysis using flow cytometry. (**B**), The data on cell cycle distribution of SCC1 and OSC19 cells after treatment with GSPs are summarized. Significant difference versus control, ^*^ *P*<0.01. (**C**), Effect of GSPs on G1 phase cell cycle regulatory proteins in SCC1 and OSC19 cells. (**D**), The effect of GSPs on the phosphorylation of pRb and E2F. The cells were treated with either vehicle alone or GSPs (20, 40, 60 µg/mL) for 48 h and thereafter harvested, cell lysates prepared and subjected to western blot analyses for cyclins, Cdks, pRb and E2F proteins.

As we found a significant growth inhibitory effect of GSPs on HNSCC cells, we determined the possible inhibitory effect of GSPs on cell cycle progression. To determine the possible mechanisms of the anti-proliferative effects of GSPs, cell cycle analysis was undertaken using SCC1 and OSC19 cell lines treated with 20, 40 or 60 µg/mL of GSPs (Figure 2A), and resultant data are summarized in Figure 2B. Treatment of SCC1 cells with GSPs for 48 h resulted in a significantly higher percentage of cells in the G0-G1 phase at all the concentrations used: 20 µg/mL (56.3%, *P*<0.05), 40 µg/mL (65.2%, *P*<0.01) and 60 µg/mL (78.7%, *P*<0.001) as compared to the non-GSPs-treated controls (46.6%). At the 24 h time-point, the effect of GSPs on G0–G1 cell cycle arrest was significantly less than that observed after treatment for 48 h. Similar results were obtained on analysis of the effects of GSPs treatment on cell cycle progression of OSC19 cells, as data are presented and summarized in Figure 2A and 2B. These data suggest that inhibition of cell proliferation/viability or induction of cell death in HNSCC cell lines by GSPs may be associated with the induction of G0–G1 arrest.

### GSPs decrease the expression of G1 phase regulatory proteins in HNSCC cells

As it has been shown that cyclins, Cdks and Cdk inhibitors play crucial roles in the regulation of cell cycle progression [29], we determined the effect of GSPs on cell cycle regulatory proteins. The results of western blot analysis revealed that treatment of SCC1 and OSC19 cells with varying concentrations of GSPs for 48 h resulted in a reduction in the expression of cyclins D1 and D2 in a dose-dependent manner (Figure 2C). Similarly, a pronounced reduction in the expression of Cdk2, Cdk4 and Cdk6 was observed in both SCC1 and OSC19 cell lines after 48 h of treatment with GSPs (Figure 2C). Further, as Cdks are known to phosphorylate pRb, releasing E2F from the cytoplasm, which leads to activation of downstream targets of E2F responsible for cell cycle regulation, we checked the effect of GSPs on the expression of pRb and E2F in SCC1 and OSC19 cells. As shown in Figure 2D, treatment of cells with GSPs induce hypophosphorylation of Rb and reduced expression of E2F1 and E2F2 in both cell lines. GSPs also reduced the expressions of PCNA and cyclin D1 compared to non-GSPs-treated control cells, which are the downstream targets of E2F and play a role in cell cycle regulation.

### GSPs up-regulate or reactivate the expression of Cdk inhibitory proteins Cip1/p21 and Kip1/p27 in HNSCC cells

**Figure 3:**
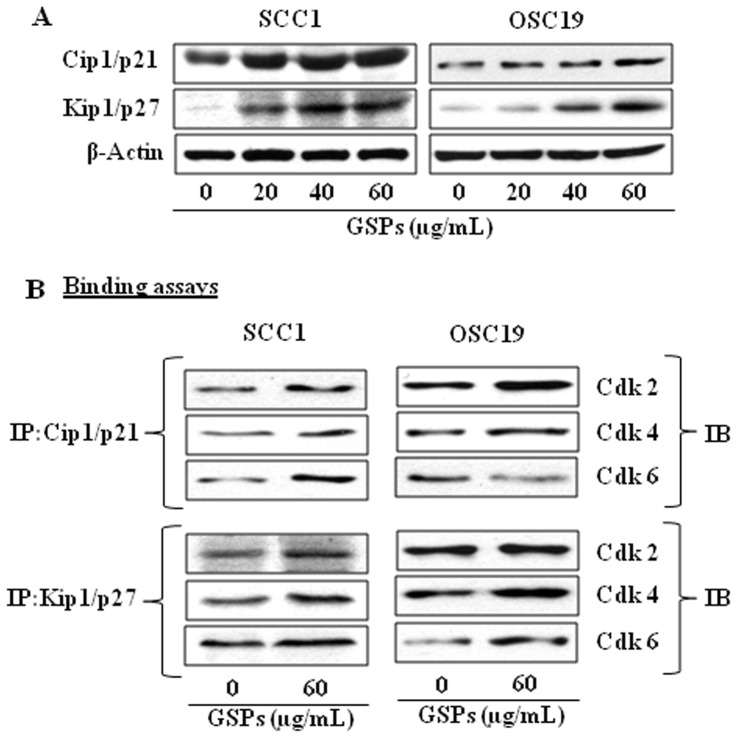
Effect of GSPs on the expression of Cdk inhibitory proteins. (**A**), Cells were treated with GSPs as detailed under Figure 2. Cell lysates were subjected to the analysis of Cip1/p21 and Kip1/p27 proteins. (**B**), Binding assay of Cip1/p21 and Kip1/p27 proteins with Cdks was performed in SCC1 and OSC19 cells. In binding assays, Cip1/p21 and Kip1/p27 were immunoprecipitated using protein-specific antibody from total protein lysates followed by western blot analysis for Cdk2, Cdk4 and Ckd6, as detailed in Materials and Methods. IP, immuno-precipitation; IB, immunoblotting.

The Cdk inhibitors, such as Cip1/p21 and Kip1/p27 proteins, regulate the progression of cells in the G0–G1 phase of the cell cycle and induction of these proteins causes a blockade of the G1 to S phase transition, thereby resulting in a G0–G1 phase arrest of the cell cycle [30]. The loss of functional Cdk inhibitors in human cancers can lead to uncontrolled cell proliferation due to an increase in the levels of the Cdk-cyclin complex [31]. As the treatment of SCC1 and OSC19 cells with GSPs resulted in G0–G1 arrest, we next examined the effect of GSPs on Cdki proteins. The data from western blot analysis revealed that treatment of SCC1 and OSC19 cells with GSPs for 48 h resulted in increased expressions of Cip1/p21 and Kip1/p27 proteins in a dose-dependent manner (Figure 3A).

The Cdk inhibitory proteins suppress cell cycle progression by binding to, and inhibiting, the kinase activity of the Cdk-Cyclin complex [29], [32], [33]. Therefore, we examined whether GSPs promote the interaction between Cdki and Cdks. To assess this act of GSPs, Cip1/p21 and Kip1/p27 were immunoprecipitated from total cell lysates and their binding of Cdk2, Cdk4 and Cdk6 assessed using western blotting. As compared to vehicle treated controls, treatment of both SCC1 and OSC19 cells with GSPs was found to increase the binding of Cdk2, Cdk4 and Cdk6 with Cip1/p21 and Kip1/p27 (Figure 3B). These observations suggest that the GSPs-induced enhancement of the levels of Cip1/p21 and Kip1/p27 and their binding with Cdks plays an important role in the GSPs-induced G0–G1 arrest of cell cycle progression in HNSCC cells.

### GSPs induce apoptosis in HNSCC cells

**Figure 4:**
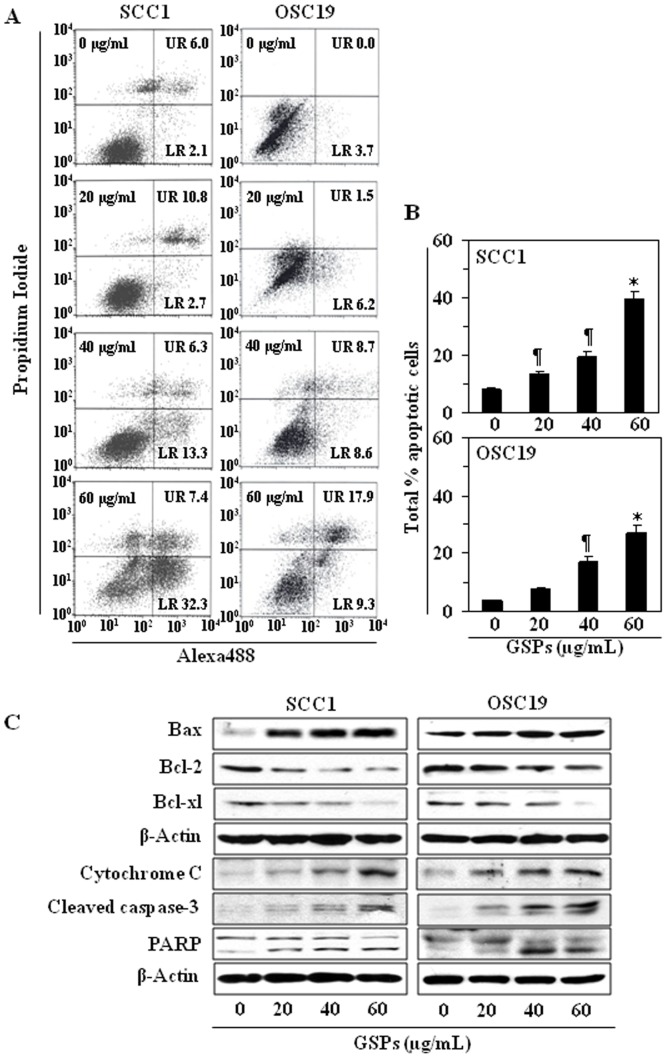
GSPs induce apoptosis in HNSCC cells in a dose-dependent manner. (**A**), SCC1 and OSC19 cells were treated with varying concentrations of GSPs (0, 20, 40 and 60 µg/mL) for 48 h, then harvested for the analysis of apoptotic cells by FACS using the Annexin V-Alexa Fluor488 Apoptosis Vybrant Assay Kit (Alexa488) following the manufacturer's protocol. The lower right (LR) quadrant of the FACS histograms indicates the percentage of early apoptotic cells and the upper right (UR) quadrant indicates the percentage of late apoptotic cells. (**B**), Total percentages (early+late stages) of apoptotic cells in each treatment group are summarized with data presented as the mean±SD of two experiments. Significant difference *vs.* non-GSPs-treated control group, ^*^ *P*<0.001; ^¶^ *P*<0.05. (**C**), Treatment of cells with varying concentrations of GSPs for 48 h results in a dose-dependent reduction in the expression levels of Bcl-2 and Bcl-xl while increasing the expression of Bax. GSPs also increase the levels of cytochrome *c* and activation of caspase-3 and PARP. β-actin was used to verify equal loading of the protein samples.

To examine whether the GSPs-induced loss of the cell viability and induction of G0/G1 phase arrest in HNSCC cells was associated with the induction of apoptosis, SCC1 and OSC19 cells were treated with GSPs for 48 h and the percentage of apoptotic cells were determined using the Annexin V-conjugated Alexa Fluor-488 (Alexa488) Apoptotic Detection Kit as previously described [26]. Apoptotic cell death was determined in terms of early- and late-stage apoptotic cells, which are shown respectively in the lower right (LR) and upper right (UR) quadrants of the FACS histograms (Figure 4A). Treatment of both the SCC1 and OSC119 cells with GSPs for 48 h resulted in a significant induction of apoptosis in both cell lines. The percentages of total apoptotic cells (in UR+LR quadrants) in SCC1 cells after GSPs treatments were as follows: 8.1% (vehicle-treated control), 13.5% (20 µg/mL, *P*<0.05), 19.6% (40 µg/mL, *P*<0.01), and 39.7% (60 µg/mL, *P*<0.001), as also summarized in Panel B. Similar results were obtained on GSPs treatment of OSC19 cells (7.7%–27.2%, as compared with control) for 48 h, as shown in Figure 4A and 4B. Treatment of cells with GSPs for 24 h did not induce significant percentage of apoptosis (data not shown).

### GSPs affect the protein expression of Bcl-2 family and activate caspase-3 and poly(ADP-ribose) polymerase (PARP) in HNSCC cells

The proteins of the Bcl-2 family play important roles in regulation of apoptosis by functioning as promoters or inhibitors of cell death process [34], [35]. Therefore, we examined the effect of GSPs on the proteins of Bcl-2 family in both SCC1 and OSC19 cells. For this purpose, SCC1 and OSC19 cells were treated with GSPs (0, 20, 40, 60 µg/mL) for 48 h, and cell lysates were prepared for western blot analysis. The data from western blot analysis revealed that treatment of cells with GSPs resulted in a dose-dependent reduction in the levels of the anti-apoptotic proteins (Bcl-2 and Bcl-xl) with a concomitant increase in the levels of pro-apoptotic protein Bax compared with the cells that were not treated with GSPs (Figure 4C). These data indicate that GSPs treatment can alter the protein levels of key members of the Bcl-2 family in a manner that favors an increase in the ratio of Bax:Bcl-2, which may contribute to the susceptibility of cancer cells to GSPs-induced apoptosis (Figure 4C) [36].

An early event in apoptosis is the disruption of the mitochondrial membrane potential. This event includes translocation of Bax from the cytosol to the mitochondria, triggers release of cytochrome c and other apoptogenic molecules from the mitochondria to the cytosol [37], [38]. In turn, these molecules contribute to the activation of caspases and subsequent cell death. Western blot analysis revealed that treatment of SCC1 and OSC19 cells with GSPs enhanced the release of cytochrome *c* into the cytosol in a dose-dependent manner (Figure 4C). GSPs also increased the activation or cleavage of caspase-3 and PARP when compared with the cells which were not treated with GSPs (Figure 4C).

### GSPs down-regulate the expression of EGFR in HNSCC cells

**Figure 5:**
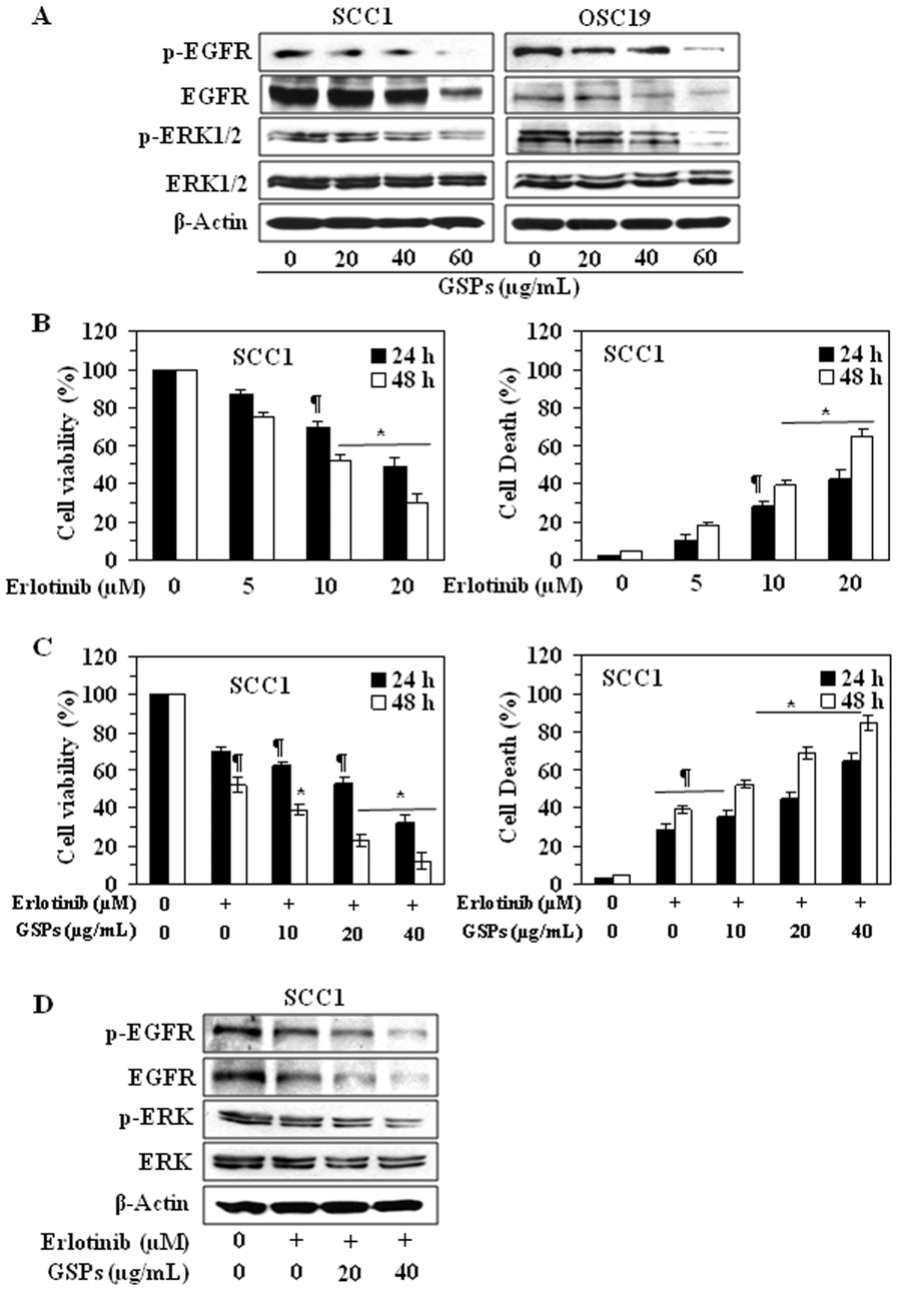
Erlotinib treatment suppresses the cell viability and the levels of EGFR in HNSCC cells. (**A**), Treatment of SCC1 and OSC19 cells with GSPs decreases the overexpression of EGFR and its downstream target ERK1/2 in these cells, as determined by western blot analysis. Treatment of cells with GSPs was similar as detailed under Figure 2. (**B**), Treatment of SCC1 cells with erlotinib, an inhibitor of EGFR, reduces cell viability (left panel) and induces cell death (right panel) in a dose- and time-dependent manner. The data on cell viability and cell deaths are expressed in terms of percent of control cells (non-erlotinib treated) as the mean±SD of 8 replicates. Significant difference *vs.* control group, ^*^ *P*<0.001; ^¶^ *P*<0.05. (**C**), Combined effect of GSPs and erlotinib (10 µM) on cell viability (left panel) and cell death (right panel) of SCC1 cells. Combined treatment of SCC1 cells with erlotinib and GSPs significantly reduces the cell viability (left panel) and increases cell death (right panel). (**D**), Combined effect of erlotinib (10 µM) and GSPs on the levels of EGFR and ERK1/2 after treatment of SCC1 cells for 48 h. Representative western blots are shown from three separate experiments.

As EGFR is overexpressed in approximately 90% of HNSCC tumors [10]–[12], we examined the effect of GSPs on the levels of EGFR and its down-stream target ERK1/2 in HNSCC cells. SCC1 and OSC19 cells were treated with various concentrations of GSPs (0, 20, 40 and 60 µg/mL) for 48 h, and cell lysates were subjected to western blot analysis of EGFR and ERK1/2. Our data revealed that treatment of both SCC1 and OSC19 cells with GSP for 48 h resulted in a dose-dependent decrease in the expression levels of total as well as phosphorylated EGFR and also reduced the phosphorylation of ERK1/2 (Figure 5A).

### Erlotinib reduces cell viability and induces cell death of HNSCC cells

Again, as EGFR is overexpressed in approximately 90% of HNSCC tumors [10]–[12], we checked the effect of erlotinib, an EGFR-targeting small molecule tyrosine kinase inhibitor, on the expression of EGFR in HNSCC cells. For this purpose, we selected SCC1 cell line as a representative of HNSCC. On treatment of SCC1 cells with varying concentrations of erlotinib for 48 h, a significant (*P*<0.05 to *P*<0.001) dose-dependent reduction in cell viability was observed (Figure 5B, left panel) and simultaneously the percentage of cell death was increased compared with non-erlotinib-treated control cells (Figure 5B, right panel).

Combination of drugs is most widely used in treating the most dreadful diseases, like cancer, and the purposes are to achieve additive or synergistic therapeutic effect, dose and toxicity reduction, and to minimize or delay the induction of drug resistance. Therefore, we also examined the combined effect of GSPs and erlotinib. The results from cell viability and cell death assay revealed that combined effect of GSPs and erlotinib (10 µM) is greater than any individual agent and the effect was dose-dependent, as shown in Figure 5C. Western blot analysis also confirmed that combined treatment of SCC1 cells with GSPs and erlotinib for 48 h resulted in decreased expressions of EGFR, p-EGFR and p-ERK1/2 proteins and it was additive effect (Figure 5D).

### Dietary GSPs inhibit the growth of HNSCC tumor xenograft

**Figure 6:**
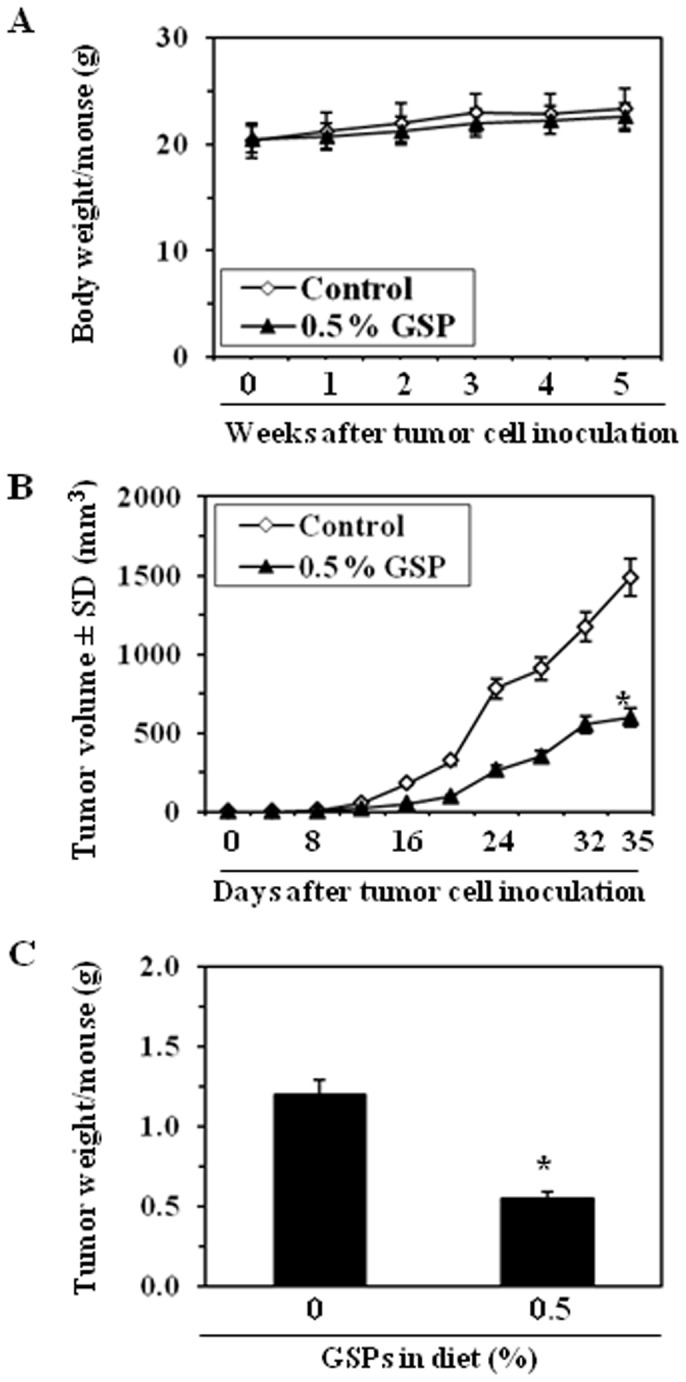
Dietary GSPs (0.5%, w/w) inhibit the growth of SCC1 tumor xenografts in athymic nude mice. (**A**), Average body weight/mouse per week in different treatment groups was recorded. (**B**), Tumor volume/mouse as mean±SD/mouse (mm^3^) in each treatment group was recorded on weekly basis. (**C**), Tumor xenograft tissues were harvested at the termination of the experiment and the wet weight of the tumor/mouse in each group is reported in grams as the mean±SD. Statistical significance vs tumors from control group, ^*^ *P*<0.001, n = 8.

Next, we sought to determine whether dietary administration of GSPs inhibits *in vivo* xenograft growth of HNSCC cells using athymic nude mouse model. Again, we selected SCC1 cell line as a representative of HNSCC cell lines for these *in vivo* studies. In our earlier *in vivo* studies with GSPs, we have found that administration of GSPs at the dose of 0.5% (w/w) in diet significantly decreased the growth of various tumors [18], [19], [21]–[23], therefore, we selected this dose of GSPs for the current *in vivo* study with HNSCC. The mice were given the AIN76A control diet alone or the same diet supplemented with GSPs (0.5%, w/w) and the effects on the growth of xenograft tumors were monitored on weekly basis. The body weights of the GSPs-treated and non-GSPs-treated nude mice were measured on weekly basis, which remained identical throughout the duration of the experiment (Figure 6A). At the same time, the mice that were given GSPs in their diet did not exhibit any physical sign of toxicity or abnormal behavior (data not shown). These observations suggest that dietary GSPs at the concentration used in these studies are non-toxic to mice.

Periodic measurement of the tumor volume indicated that the average growth of tumor xenograft in terms of total tumor volume/mouse was higher in the non-GSPs-treated control mice than the GSPs-treated groups throughout the duration of the experiment. As shown in Figure 6B, the mice which received GSPs in their diet resulted in 61% (*P*<0.001) less tumor volume at the termination of the experiment at 35^th^ day compared to non-GSPs-fed mice. At the termination of the experiment, the mice were sacrificed, the tumors harvested, and the weight of wet tumor/mouse in each treatment group was recorded. The wet weight of the tumors in GSPs-fed mice was 51% lower (*P*<0.001) as compared to the tumors from non-GSPs-fed mice (Figure 6C).

### Dietary GSPs inhibit the expressions of Go–G1 regulatory proteins and increase Cdk-Cdki binding in tumor xenograft tissues of HNSCC

**Figure 7:**
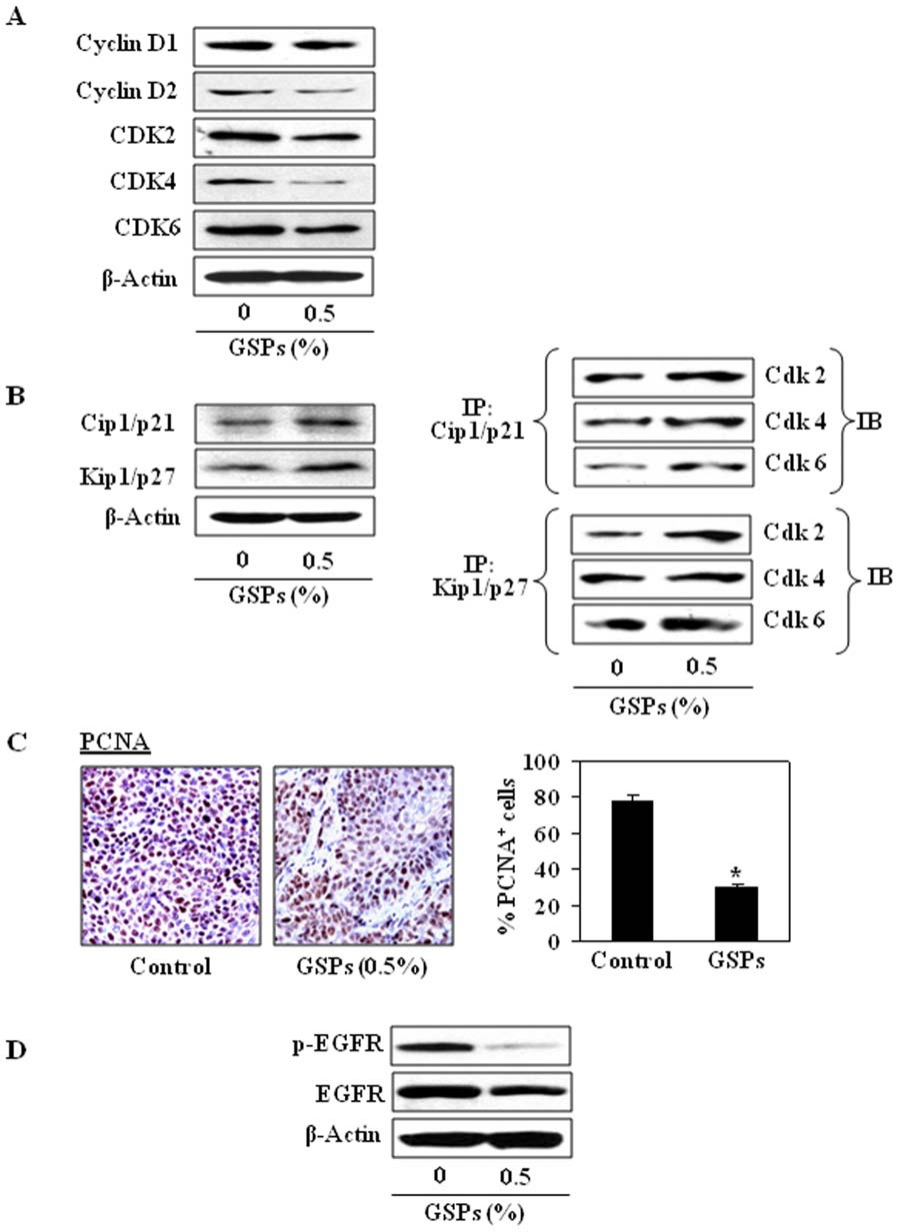
Effect of dietary GSPs on cell cycle regulatory proteins in SCC1 tumor xenograft samples. (**A**), GSPs inhibit the expressions of cyclins and Cdks proteins in tumor xenograft samples compared to controls. Tumor lysates were subjected to western blot analysis. (**B**), GSPs enhance the levels of Cdk inhibitory proteins, Cip1/p21 and Kip1/p27, in tumors. Binding assays of Cip1/p21 and Kip1/p27 proteins with Cdks was performed in SCC1 tumor xenograft tissues using western blot analysis. GSPs enhanced the binding of Cip1/p21 and Kip1/p27 proteins with Cdks. IP, immuno-precipitation; IB, immunoblotting. (**C**), Immunohistochemical detection of proliferating cells in tumor xenograft tissues. PCNA-positive cells were identified as an estimate of the proliferation index (left panel), and data are presented in terms of percent PCNA-positive cells from 5 tumor xenograft samples (right panel). Statistical significance *vs*. tumor samples from mice receiving the control diet only, ^*^ *P*<0.001. (**D**), GSPs decrease the expressions of EGFR and p-EGFR in growing tumor xenograft samples.

To verify the data obtained in *in vitro* system, we examined the effect of dietary GSPs on cell cycle regulatory proteins *in vivo* tumor xenograft model. For this purpose, tumor xenograft samples from GSPs-fed mice and from mice that received the control diet were analyzed using western blot analysis. Dietary administration of GSPs resulted in a marked decrease in the expression levels of cyclins (Cyclin D1 and cyclin D2) and Cdks (Cdk2, Cdk4, and Cdk6) compared to the tumors from non-GSPs-fed mice, as shown in Figure 7A. As the Cdki regulate the progression of cells in the G0-G1 phase of the cell cycle, we checked the effect of GSPs on the levels of Cdk inhibitory proteins in xenograft samples. Western blot analysis revealed that dietary GSPs increased the protein expression of Cip1/p21 and Kip1/p27 compared to the tumor xenograft samples from non-GSPs-fed mice (Figure 7B). Further, GSPs increased the binding of Cdk2, Cdk4 and Cdk6 with Cip1/p21 and Kip1/p27 (Figure 7B). These results further suggest that an increased interaction between Cdki with Cdks plays an important regulatory role in the GSPs-induced G1 arrest of cell cycle progression and finally contributing to the suppression of the growth of SCC1 tumor xenografts.

The results of the immunohistochemical analysis of PCNA-positive cells in tumor xenograft tissues indicated that the percentage of proliferating cells was significantly lower (60%, *P*<0.01) in tumor xenografts from GSPs-treated mice than in the tumor xenografts from the control mice (Figure 7C, left panel). Resultant data on PCNA-positive cells in both groups are summarized (right panel). Western blot analysis also revealed that in mice that were administered GSPs in diet the levels of EGFR and p-EGFR were decreased in tumor xenograft tissues compared with the tumor xenograft samples of those mice which were not given GSPs in diet (Figure 7D).

### Dietary GSPs enhance apoptotic cell death of HNSCC cells in tumor xenografts

**Figure 8:**
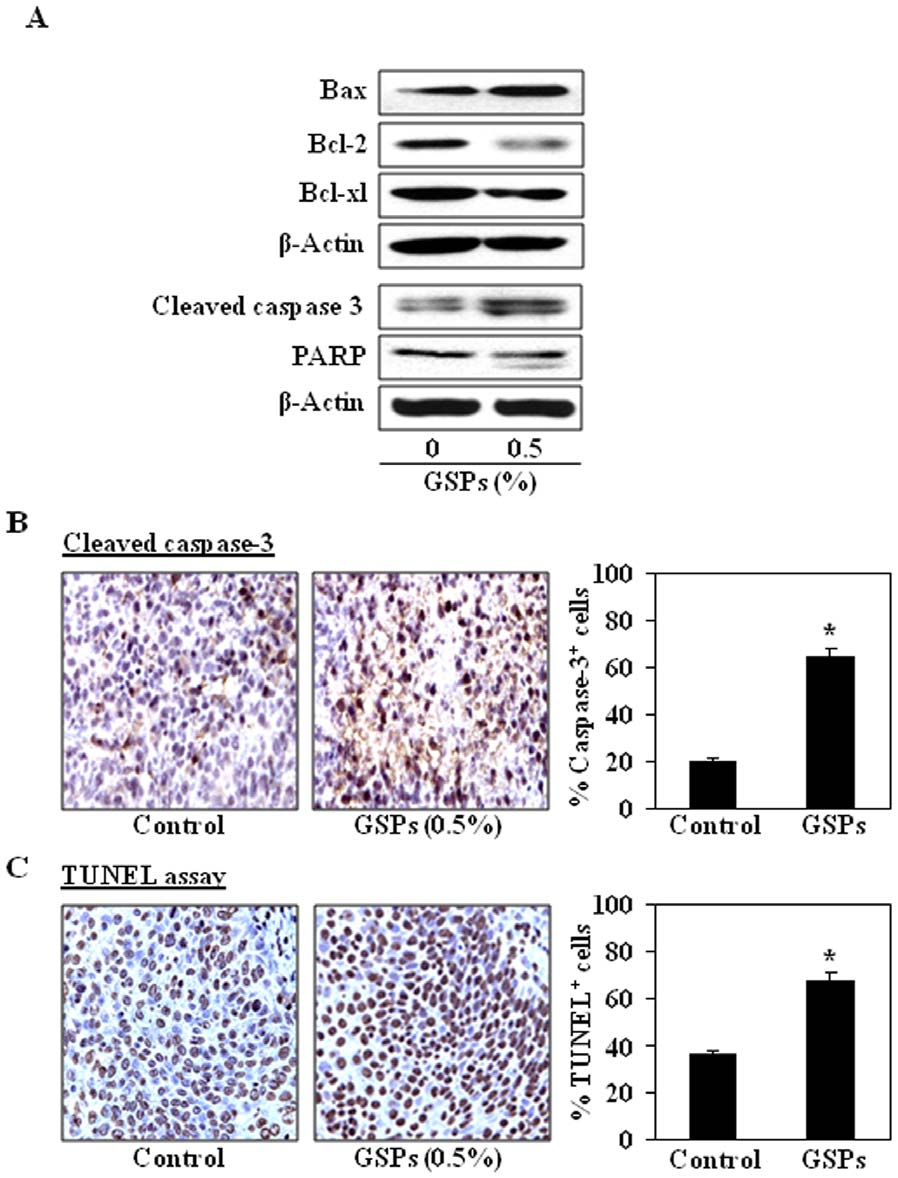
Dietary GSPs induce apoptosis in tumor xenograft cells. (**A**), GSPs inhibit the expression of anti-apoptotic proteins while increase the expressions of pro-apoptotic protein in SCC1 xenograft tissues. GSPs also enhance the activation of caspase-3 and PARP proteins in tumor xenografts compared to controls. Tumor lysates were subjected to the analysis of protein levels using western blot analysis. Representative blots are presented from the independent experiments from 4 different tumors from each treatment group. (**B**), Immunohistochemical detection of activated caspase-3-positive cells. Activated caspase-3-positive cells were counted, summarized and presented in terms of percentage of total cells as the mean±SD (right panels). (**C**), Immunohistochemical detection of TUNEL-positive cells was performed in tumor xenograft tissues from GSPs-treated and non-GSPs-treated mice (left panels). TUNEL-positive cells were counted, summarized and presented in terms of percentage of total cells as the mean±SD (right panels). Summarized data are presented from tumor xenograft samples of 5 mice/group.

To examine whether inhibition of xenograft growth by dietary GSPs is due to the death of cells in SCC1 xenograft tissues, the xenograft tumors were subjected to the analysis of pro- and anti-apoptotic proteins of the Bcl-2 family using western blot analysis. As shown in Figure 8A, the levels of the anti-apoptotic proteins, Bcl-2 and Bcl-xl, were lower in the SCC1 xenograft tumors from mice treated with GSPs than in the tumors in control mice that did not receive GSPs, whereas the levels of the pro-apoptotic protein, Bax, were higher. The cleavage of caspase-3 and PARP proteins was higher in the tumor samples from the GSPs-treated mice than the control mice (Figure 8A). Immunohistochemical detection of activated caspase-3-positive cells in tumor xenograft samples revealed that the numbers of caspase-3-positive cells were higher in tumors from GSPs-fed mice than in the tumors of non-GSPs-fed mice. The percentage of activated caspase-3-positive cells in tumor samples from mice administered GSPs was higher (61%, *P*<0.01) than the percentage of caspase-3-positive cells in the tumors of mice that did not receive GSPs (20%) (Figure 8B). Apoptotic index in tumor samples was further verified using a TUNEL assay. Immunohistochemical analysis revealed significantly higher numbers of TUNEL-positive cells in the samples of xenografts from the group of mice administered GSPs (*P*<0.01) as compared with the numbers in the samples of xenografts from the mice that did not receive GSPs in diet (Figure 8C, left panel). The numbers of TUNEL-positive cells in the GSPs-treated and non-GSPs-treated tumors were counted and resultant data are summarized in Figure 8C (right panel).

## Discussion

The anti-tumor activity of GSPs has been shown in some preclinical models [18]–[24]; however the anti-tumor activity of GSPs has not been explored in HNSCC. We therefore examined the effects of GSPs on various HNSCC cell lines using both *in vitro* and *in vivo* models. Here, we report that GSPs significantly reduce the viability and induce cell death/apoptosis of various cell lines of human HNSCC, which were derived from oral cavity (SCC1), larynx (SCC5), pharynx (FaDu) and tongue (OSC19), suggesting that GSPs may have a beneficial therapeutic effect on HNSCC. Importantly, GSPs did not exhibit toxicity to non-neoplastic human bronchial epithelial cells under identical conditions used in this study.

Control of cell cycle progression in cancer cells is considered to be an effective strategy for the control of tumor growth [29], [30], and studies have revealed that cell cycle regulators are frequently mutated in most common human malignancies [39], [40]. Our *in vitro* data indicated that treatment of HNSCC cells with GSPs resulted in G0–G1 phase arrest of cell cycle progression which indicates that one of the mechanisms by which GSPs may act to inhibit the proliferation of HNSCC cells is inhibition of cell cycle progression. Our finding of a marked decrease in the levels of cyclins and Cdks in SCC1 and OSC19 cells on GSPs treatment suggests the disruption of the uncontrolled cell cycle progression of these cells, and that the GSPs-induced G1 phase arrest is mediated through the upregulation of Cdki proteins Cip1/p21 and Kip1/p27, which increases the formation of heterotrimeric complexes with the G1/S Cdks and cyclins thereby inhibiting their activity. Intriguingly, the SCC1 cells do not express p53 or express at extremely low level [41], the upregulation of p21 by GSPs in this cell line appears to be by p53-independent pathway. As induction of tumor suppressor proteins (e.g., p21) induce downstream inhibition of cyclin D1 and hypophosphorylation of Rb, leading to inhibition of E2F function and G1 phase arrest, we checked the effect of GSPs on this pathway. We observed that GSPs induce hypophosphorylation of Rb, inhibition of E2F levels in both cell lines, which lead to downregulation of PCNA and cyclin D1 and finally G1 phase arrest. This act of GSPs may be one of the possible mechanisms of anti-carcinogenic effect in HNSCC cells. The increased expression of G1 cyclins in cancer cells provides an uncontrolled growth advantage because most of these cells either lack Cdki or the expression of Cdki is not at a sufficient level to control Cdk-cyclin activity [33]. G1 phase arrest of cell cycle progression provides an opportunity for cells to either undergo repair mechanisms or follow the apoptotic pathway. Our flow cytometry data indicate that treatment of HNSCC cells with GSPs resulted in significant induction of apoptosis. Apoptosis plays a crucial role in eliminating the mutated neoplastic and hyper-proliferating neoplastic cells from the system, and therefore is considered as a protective mechanism against cancer progression [42]. Acquired resistance toward apoptosis is a hallmark of most and perhaps all types of cancer. Therefore, GSPs seem to be a potent chemotherapeutic agent for HNSCC.

Commonly, the induction of apoptosis in cancer cells involves the up-regulation of pro-apoptotic proteins and/or down-regulation of anti-apoptotic proteins. A major apoptotic signal transduction cascade associated with induction of apoptosis includes the proteins of Bcl-2 family, which either promote cell survival or promote apoptosis [43], [44]. We found that treatment of SCC1 and OSC19 cells with GSPs resulted in a dose-dependent decrease in the levels of anti-apoptotic proteins and a simultaneous increase in the pro-apoptotic protein. It is well known that the release of cytochrome *c* from mitochondria into cytosol is an important step in the apoptotic mechanism, which leads to the activation of caspases and PARP. Once cytochrome *c* located in cytosol, forms multi-proteins complex with Apaf-1 and procaspase-9, and initiates caspase-3 activation, leading to cell apoptosis. Our data suggests that GSPs-induced apoptosis in HNSCC cells is mediated through the release of cytochrome *c* from mitochondria and subsequently activation of caspase-3 and PARP, and this may be a possible mechanism of GSPs-induced apoptosis in HNSCC cells.

As pointed out earlier, EGFR is over expressed in most of the HNSCC and its inhibition indicates therapeutic effect in head and neck cancer clinically. Our study demonstrates that treatment of SCC1 and OSC19 cell lines with GSPs decreases the expression levels of total EGFR as well as p-EGFR and its downstream target ERK1/2. Thus it can be suggested that inhibition of cell proliferation by GSPs is mediated, at least in part, through the down-regulation of EGFR/ERK signaling pathway. To further verify that GSPs inhibit the proliferation potential or cell viability of HNSCC cells through their inhibitory effects on EGFR/ERK pathway, SCC1 cells were treated with the erlotinib, an inhibitor of EGFR, and its effect on cell viability and cell death was evaluated. It was observed that similar to the action of GSPs, erlotinib also reduces cell viability and induces cell death in SCC1 cells. Additionally, the combined effect of GSPs and erlotinib on inhibition of cell viability of SCC1 cells was more than any individual agent.

The *in vitro* cell culture models are a good system for preliminary screening of the effects of chemotherapeutic agents; the observations must be verified *in vivo* animal models prior to their potential consideration for their use in humans. Therefore an *in vivo* model of tumor xenografts was used to verify the chemotherapeutic potential of GSPs against HNSCC cell growth. The GSPs were administered in the diet of the mice as this approach has proven effective in other cancers [18]–[24], [45]. Our study provides evidence that dietary administration of GSPs inhibits the growth of SCC1 tumor xenografts without any apparent sign of toxicity in the athymic nude mice. The identification of molecular targets is an important consideration in terms of monitoring the clinical efficacy of cancer chemopreventive or cancer therapeutic strategies in suggesting potential combinations with other agents or drugs, and in elucidating the mechanisms of action of any test agent. In this context, the inhibitory effect of dietary GSPs on the growth of tumor xenograft in athymic nude mice was studied and found to be associated with the: (i) control of cell cycle regulation, and (ii) induction of apoptotic cell death of tumor cells, as indicated by the analysis of the proteins of Bcl-2 family, TUNEL-positive and activated caspase-3-positive cells in tumor xenograft samples at the termination of the *in vivo* animal experiments.

In summary, the results of this study show for the first time the chemotherapeutic efficacy of GSPs on the growth of head and neck cancer cells *in vitro* and tumor xenograft growth *in vivo*. Thus GSPs appear to be an attractive dietary bioactive phytochemicals for the management of head and neck cancer; however, more mechanism-based studies are required *in vivo* models to further identify and verify the molecular targets and mechanism of actions of this agent.
